# Research needed on urban Indigenous health inequalities

**DOI:** 10.2471/BLT.23.290868

**Published:** 2023-12-29

**Authors:** Abdullah A Mamun, Edmund Wedam Kanmiki, Stuart Leske, Janet Stajic, James Ward

**Affiliations:** a UQ Poche Centre for Indigenous Health, The University of Queensland, Brisbane, 74 High Street Toowong, Queensland, 4066, Australia.; Correspondence to Abdullah A Mamun (email: a.mamun@uq.edu.au).

Indigenous peoples are culturally distinct peoples and societies who share ties to their ancestral lands and natural resources where they live, visit or have been displaced from. An estimated 476.6 million Indigenous people, speaking over 4 000 of the world’s approximately 6 700 languages^1^, live across 90 countries worldwide.^2^ Although Indigenous people represent around 6.2% of the global population of 7.7 billion, data from 23 countries show that of 6 189 651 people surveyed and living in extremely poor conditions, an estimated 18.7% (1 157 465) are Indigenous people.^1^ This group faces higher health inequalities including greater health risks, suboptimal health outcomes and greater unmet needs for health and social services.^3^ Globally, the life expectancy of Indigenous people is 20 years lower than that of non-Indigenous people.^4^ Colonization created systems and structures where Indigenous peoples often experience racism, discrimination, marginalization and exclusion, subsequently having poor access to national health systems, water and sanitation, housing and education. These conditions have made Indigenous peoples extremely vulnerable to the impact of natural disasters, disease outbreaks and climate change.

Increasing global urbanization has been accompanied by a rapid growth in Indigenous people living in urban areas.^5^ As noted at the sixth session of the United Nations Permanent Forum on Indigenous Issues,^6^ the increasing urbanization of Indigenous people requires developing culturally specific policies enabling culturally appropriate childhood education, health and provisions for maintaining Indigenous communities, connections to country and cultural identities.

**Fig. 1:**
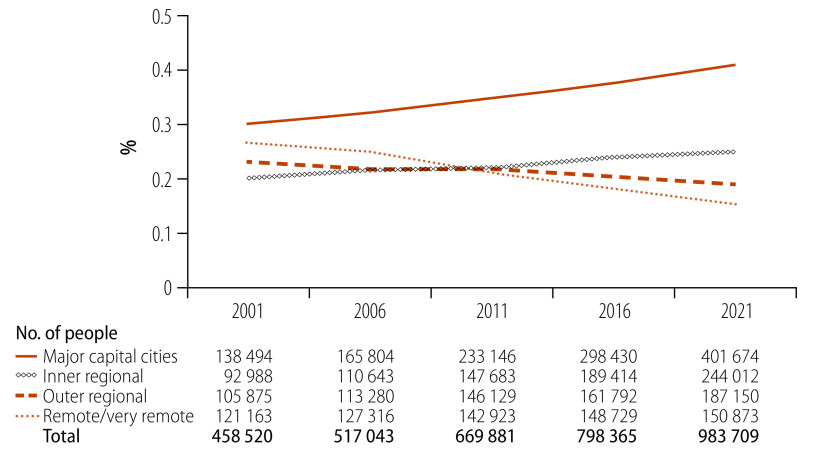
Area of residence of Indigenous population, Australia, 2001–2021

Note: Remote area includes both remote and very remote areas.

Indigenous people in Australia, comprising Aboriginal and Torres Strait Islander peoples, are the oldest continuous culture on earth; as an assertion of their sovereignty, they continue to pursue self-determination and cultural preservation through initiatives that are embedded in cultural understandings of Indigenous ways of knowing, being and doing.^4^ The 2021 Australian Population Census estimated the population of Aboriginal and Torres Strait Islander people at 983 709.^7^ Although representing just under 4% of Australia’s total population,^7^^,^^8^ this population has experienced major shifts in urbanization, particularly in the last two decades. The proportion of Indigenous people residing in major cities of Australia increased from 30% (138 494/458 520) in 2001 to 41% (401 674/983 709) in 2021, and conversely declined from 26% (121 163/458 520) in 2001 to 15% (150 873/983 709) in 2021 in remote and very remote areas (Fig. 1). Multiple factors contribute to the urbanization of Indigenous people, including ongoing dispossession of traditional lands, natural disasters induced by climate change, as well as perceptions of better opportunities in education, health, employment, social amenities and civil society participation in cities.^3^^,^^4^^,^^5^

Data sources: Australian Bureau of Statistics Censuses of 2001, 2006, 2011, 2016 and 2021.

Although 41% of Indigenous people in Australia live in major capital cities, this population accounts for 56% of the total disease burden (139 902/249 656 disability-adjusted life years), 61% of the non-fatal burden (77 706/126 547 years lived with disability) and 51% of the fatal burden (62 196/123 108 years of life lost), despite having physical access to health and well-being services.^8^ The difference in life expectancy for the Indigenous and the non-Indigenous populations in major cities of Australia for the period 2020–2022 was 8.5 years for males and 7.5 years for females.^9^ Urban Indigenous people also experience the highest levels among the Australian population of health risk factors including smoking, alcohol and substance abuse, sleep disorders, lower levels of formal education, higher unemployment rates and incarcerations. Many of these indicators are related to intergenerational trauma as well as systems and structures that fail Indigenous peoples in these cities,^8^ compounded by a dearth of policy, research and programmes focused on urban Indigenous people.

A pervasive discourse in Indigenous health is a focus on remote Indigenous communities, which we affirm is required, but this is often done at the expense of an urban Indigenous health focus, and with an assumption that urban Indigenous people can access mainstream services close to where they live.^6^^,^^8^ However, mainstream health facilities in urban areas are not adequately serving the needs of Indigenous peoples because of either a lack of Indigenous cultural capability, absence of Indigenous staffing, unwelcoming environments, racism or classism.^8^^,^^10^ Thus, while addressing health equity gaps in remote areas is needed, growing urbanization means more efforts are required to address this inequity and across all geographic areas to improve the health and well-being of Indigenous people.^11^

Aboriginal and Torres Strait Islander community controlled health services are a major service provider for Indigenous people in Australia through comprehensive primary health-care clinics administered and governed by local Aboriginal communities. The services were established to address the many unmet health-care needs of Indigenous people. These services are a practical demonstration of self-determination for Indigenous peoples in Australia, and in 2023, more than 150 service providers exist in most areas of Australia, providing culturally safe and affirmative holistic primary health care for their clients.

When they are adequately resourced and implemented, Indigenous-led and research-informed systems of care have proven effective in addressing health inequity in urban areas, by improving health-care access, utilization and outcomes.^12^ One such example is the Birthing in Our Community service operating on the lands of the Yuggera and Turrbal people, in Meanjin, the city now called Brisbane. 

The main elements of the Birthing in Our Community model include an Indigenous-led, developed and implemented partnership-based model of governance and service operation. The model offers continuity of provision of midwifery care throughout pregnancy, birthing and post-natal periods, as well as continuity of Indigenous Family Support Practitioner, a unique role that involves engaging and supporting families during the pregnancy and working with the family to manage critical social, economic and cultural determinants of health. The model has a community-based hub, a safe and welcoming place for Aboriginal and Torres Strait Islander women, babies and families to gather.^12^ The model particularly focuses on growth and development of a skilled Indigenous birthing workforce – recognizing its importance when providing a culturally safe and responsive service and in directly targeting a key social determinant of health and well-being. Finally, the framework promotes the integration of a multidisciplinary team of psychosocial health and well-being services, and supports and facilitates access to partnership programmes and specialists.

In 2020–2021, analysis of 245 Indigenous women who delivered 249 babies in the Birthing in Our Community services indicated that 83% (203) of these women had five or more antenatal visits, and 93% (227) of women had their first antenatal visit in the first trimester.^12^ Compared to usual care in public health services, the programme was associated with 38% lower odds of preterm birth (odds ratio, OR: 0.62; 95% confidence intervals, CI: 0.42–0.93); 54% higher odds of adequate antenatal care visits (OR:1.54; 95% CI: 1.13–2.09); and 34% higher odds (OR:1.34; 95% CI: 1.06–1.70) of exclusive breastfeeding.^13^ Initially established with a grant from the National Health and Medical Research Council, the programme has become permanent in South East Queensland. Indigenous clients have described feeling a sense of belonging, having multiple children through the service, and feeling supported by the birthing clinic specialists throughout these pregnancies.^12^

Another programme, Deadly Choices,^14^ is also led by the community-controlled health services as a social marketing campaign that the Institute for Urban Indigenous Health initiated in South East Queensland in 2010. The word “deadly” is a contranym, meaning awesome or fantastic, and is used as praise in Aboriginal English.^15^ One component of the Deadly Choices campaign entails increasing the number of Indigenous Health Checks, a federally funded annual health check. After adopting Deadly Choices, Mamu Community Controlled Health Service in Innisfail, Queensland, saw increases in health checks from 904 checks in 2014–2015 to 1603 checks in 2016–2017, a 77% increase.^14^

Similarly, in Inala in Brisbane, the Inala Indigenous Health Service is a culturally safe public health-care service that has been led by an Indigenous medical doctor for over 20 years. The number of Aboriginal and Torres Strait Islander patients attending this service increased from 12 in 1994 to over 10 000 by 2014.^16^ The Inala Indigenous Health Service eventually expanded to become the Southern Queensland Centre of Excellence in Aboriginal and Torres Strait Islander Primary Health Care.^16^

The development, implementation and evaluation of all such services are examples of what is needed to address ongoing urbanization of Indigenous peoples. Yet Australia still lacks a focus on urban Indigenous health policy and commitment to this area including a national research agenda. An Indigenous-led urban health research agenda that centres Indigenous sovereignty and self-determination is needed to address the urban health disparities in urban Indigenous populations.

The Poche Centre for Indigenous Health at the University of Queensland is an Indigenous-led research centre that works collaboratively with the Institute for Urban Indigenous Health and several community-controlled health services in state capital cities of Australia. The centre, in collaboration with the Institute for Urban Indigenous Health, has established a Research Alliance for Urban Community Controlled Health Services that intends to deliver an innovative national Indigenous urban health research agenda based on culturally valid methods and ethical principles to bring about transformative changes in health systems, health policy and health outcomes for urban Indigenous populations.

Globally, strategic investment and efforts must focus on urban Indigenous populations through engaging and working with Indigenous communities and community organizations – which is crucial to ensure the development of culturally safe solutions.^17^ The United Nations has called immediate attention to the development of culturally specific policies in the areas of health care, housing, education and employment to ensure that progress in these areas is led and shared by Indigenous people.^6^ To do so, we advocate for grant-funded development of local, national and international collaborative urban Indigenous health research networks to ensure focus, commitment and collaborative approaches to addressing the health and well-being needs of urban Indigenous people.

Not addressing these needs endangers closing the gap in health inequity and hinders the attainment of the sustainable development goals (SDGs), which are aimed at all-inclusiveness in leaving no one behind. The rapid urban Indigenous population growth will increase health disparities, widen the gap of inequities and jeopardize meeting the SDGs, unless Indigenous-led and research-informed systems of care are implemented to improve health-care access, utilization and outcomes. 
